# A unifying hypothesis for hydrocephalus, Chiari malformation, syringomyelia, anencephaly and spina bifida

**DOI:** 10.1186/1743-8454-5-7

**Published:** 2008-04-11

**Authors:** Helen Williams

**Affiliations:** 1 19 Elibank Road, Eltham, London, SE9 1QQ, UK

## Abstract

This work is a modified version of the Casey Holter Memorial prize essay presented to the Society for Research into Hydrocephalus and Spina Bifida, June 29^th ^2007, Heidelberg, Germany. It describes the origin and consequences of the Chiari malformation, and proposes that hydrocephalus is caused by inadequate central nervous system (CNS) venous drainage. A new hypothesis regarding the pathogenesis, anencephaly and spina bifida is described.

Any volume increase in the central nervous system can increase venous pressure. This occurs because veins are compressible and a CNS volume increase may result in reduced venous blood flow. This has the potential to cause progressive increase in cerebrospinal fluid (CSF) volume. Venous insufficiency may be caused by any disease that reduces space for venous volume. The flow of CSF has a beneficial effect on venous drainage. In health it moderates central nervous system pressure by moving between the head and spine. Conversely, obstruction to CSF flow causes localised pressure increases, which have an adverse effect on venous drainage.

The Chiari malformation is associated with hindbrain herniation, which may be caused by low spinal pressure relative to cranial pressure. In these instances, there are hindbrain-related symptoms caused by cerebellar and brainstem compression. When spinal injury occurs as a result of a Chiari malformation, the primary pathology is posterior fossa hypoplasia, resulting in raised spinal pressure. The small posterior fossa prevents the flow of CSF from the spine to the head as blood enters the central nervous system during movement. Consequently, intermittent increases in spinal pressure caused by movement, result in injury to the spinal cord. It is proposed that posterior fossa hypoplasia, which has origins in fetal life, causes syringomyelia after birth and leads to damage to the spinal cord in spina bifida. It is proposed that hydrocephalus may occur as a result of posterior fossa hypoplasia, where raised pressure occurs as a result of obstruction to flow of CSF from the head to the spine, and cerebral injury with raised pressure occurs in anencephaly by this mechanism.

The current view of dysraphism is that low central nervous system pressure and exposure to amniotic fluid, damage the central nervous system. The hypothesis proposed in this essay supports the view that spina bifida is a manifestation of progressive hydrocephalus in the fetus. It is proposed that that mesodermal growth insufficiency influences both neural tube closure and central nervous system pressure, leading to dysraphism.

## Introduction

Hydrocephalus involves degrees of raised central nervous system (CNS) pressure and extracellular fluid accumulation. It is thought to result from many unrelated disease processes. The relationship between hydrocephalus and spina bifida has been the subject of prolonged debate. The Chiari I malformation is related to posterior fossa hypoplasia and causes spinal injury in syringomyelia by obstruction to cerebrospinal fluid (CSF) flow at the foramen magnum [1,2]. The Chiari II malformation is found in association with spina bifida and is thought by many to be unrelated to Chiari I, with neural injury and reduced posterior fossa size caused by failure of neural tube closure and its consequences, including toxicity caused by exposure to amniotic fluid [3,4]. It is proposed that there is an alternative explanation for the varied manifestations of anencephaly and spina bifida that represents a combination of defective neural tube closure and hydrocephalus in the fetus. The hypothesis argues that:

• Hydrocephalus progresses because of venous insufficiency. This occurs with all primary abnormalities that cause a pathological increase in CNS pressure.

• Hindbrain herniation is caused by an abnormal cranio-cervical pressure gradient or hindbrain compression resulting from posterior fossa hypoplasia. These mechanisms frequently act together to give rise to the Chiari malformations.

• Chiari malformations cause obstruction to CSF flow that elevates CNS pressure and damages neural tissue by ischemic and mechanical forces.

• Chiari-related syringomyelia, spina bifida and anencephaly form a spectrum of disease related to restricted growth of the posterior fossa.

### Intracranial pressure

The terms pressure and volume may often be used interchangeably when describing CNS pressure, because pressure depends upon volume [5]. The hypothesis depends upon the relationship between pressure and volume, which includes the phenomenon of compliance. Compliance enables a volume increase to occur in the intrathecal CNS space without causing a pressure increase [5], and occurs when a corresponding amount of venous blood is displaced. Compliance affects the ability of the CNS to accommodate volume fluctuations that occur with movement and so avoid ischemia.

**Figure 1:**
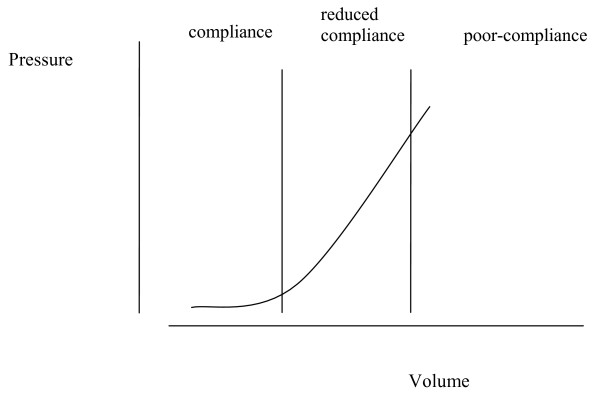
**The intracranial pressure volume relationship: a graph depicting the effect on intracranial pressure of increasing volume.** Central nervous system compliance depends upon intrathecal volume. Redrawn from [5], with permission [Additional file 1].

Blood from the CNS is drained by a venous plexus that is extensive, anastamotic and valveless. It allows blood to flow in a retrograde direction, away from the heart and into the CNS with postural movements [6,7]. CSF pressure fluctuations with movement have been observed directly [8] and small movements such as occur during speech, may cause detectable CNS pressure fluctuations [5]. Veins situated between the dura and surrounding bone are highly compressible and capable of large volume fluctuations [7]. Such veins are prominent in the spine, and are susceptible to volume fluctuation with alterations in body cavity pressure [7]. As CNS venous volume increases with retrograde flow veins may be distended due to 'back pressure' or may be compressed due to elevation of overall pressure, resistance to venous outflow then increases, outflow is reduced, and pressure will tend to rise. Pressure increase in response to increasing intrathecal volume corresponds to a phase of reduced compliance [5]. If volume increases further, a pressure may be reached which compromises arterial flow, resulting in ischemia of neural tissue [5]. The relationship between overall CNS pressure and added volume is shown in Fig. 1. The pressure volume index (PVI) is the volume that when added to the CSF space results in a ten-fold increase in pressure. The normal adult range is 13–26 ml [9]. Adults will have a larger PVI than infants because of a larger central nervous system with a correspondingly greater venous volume.

**Figure 2:**
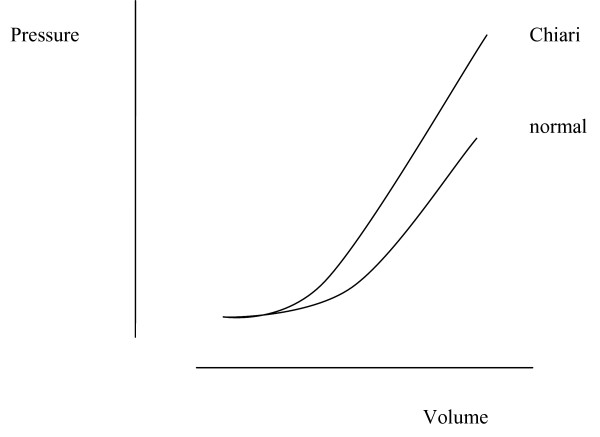
**A hypothetical graph showing the relation between spinal or cranial pressure, and venous volume, with and without Chiari malformation.** CSF obstruction at the foramen magnum divides the CSF space into cranial and spinal compartments. The sum of the pressure volume indices of the two spaces would approximate that of the unobstructed CNS. The pressure response to influx of venous volume either in the head or the spine may be enhanced by the presence of Chiari malformation.

CSF flow contributes to compliance by regulating the transluminal pressure of veins and therefore their patency and flow. Poiseuille's law states that laminar flow in a tube is inversely proportional to the fourth power of the radius. Hence a decrease in vessel diameter will cause a four-fold increase in resistance. If any pressure increase associated with increase in CNS volume is distributed throughout the intrathecal space, there will be a small average reduction in the diameter of many veins. If the whole CNS venous system is affected by a CNS volume increase, the overall reduction in venous outflow will be minimised, thus free flow of CSF facilitates venous drainage. Veins inside the head do not collapse when moving to an upright position because the movement of CSF with gravitational force allows both fluids to experience the same pressure gradient [10], maintaining the patency of veins. Obstruction to CSF flow at the foramen magnum is an important cause of reduced compliance. Movement of the body appears to contribute to foramen magnum obstruction, so that CSF flow may be affected by posture [8]. The head and spine may be transiently separated into compartments with reduced pressure volume indices. Evidence of obstruction may be present without an obvious hindbrain hernia [11,12] or with cerebellar deformity. Pressure gradients between the CSF spaces of the head and spine are normally those due to gravity [13]. In the presence of an obstruction associated with spina bifida or syringomyelia, pressure may fluctuate independently in the head and spine [8,14,15]. This phenomenon has been termed 'craniospinal pressure dissociation'. With normal CSF pathways at the foramen magnum, retrograde flow of venous blood into the CNS may be compensated by compression of distant veins. If a volume increase occurs in either the head or the spine in the presence of foramen magnum obstruction, it will, according to this hypothesis cause a pressure response which will be greater than if CSF pathways were unobstructed. It is proposed therefore that with Chiari malformation retrograde venous flow causes an enhanced pressure response, as illustrated in Fig. 2.

In a relaxed subject with normal compliance, small volume fluctuations caused by blood flow will not be detected by overall pressure measurement. As compliance is reduced, minor volume fluctuations may result in measurable pulsations and physical movement will cause the greatest pressure fluctuations and peaks. The elevation of CSF pressure in normal pressure hydrocephalus [16] and in hydrocephalic children observed during continuous pressure monitoring [17] supports the hypothesis. Reduction of pulsatility of CSF pressure following cranial expansion as a treatment for hydrocephalus [18] is also consistent with the proposed theory.

### The mechanism of extracellular fluid accumulation

It is proposed that when compliance is reduced:

• Venous volume increase with postural movements will cause delay of venous and arterial flow in the parenchyma.

• At peaks of pressure, vessels in the parenchyma of the brain or spinal cord may be sufficiently compressed to result in ischemia.

Continuous unidirectional flow in venous plexus vessels is not essential, venous blood may be diverted via anastamoses to lower resistance vessels whereas continuous flow in parenchymal vessels is necessary to provide oxygen. Full compliance benefits flow in all vessels [5,19]. The autonomic nervous system, by controlling vessel diameter and flow, is known to influence extracellular fluid volume in many tissues, with filtration or absorption taking place in venules depending on local circumstances [10]. Autonomic regulation of flow in CNS vessels is likely to influence the rate of filtration into the parenchyma. If, however, autoregulation is unable to compensate for the effect of venous pressure fluctuations, extracellular fluid will accumulate causing reduction in compliance. Consequently, venous outflow will be further compromised and hydrocephalus will progress. Ischemia is likely to be a part of the pathological process with a hyperemic response enhancing filtration of fluid into the parenchyma. Volume increases that do not cause ischemia are accommodated, but progressive volume increase will cause ischemia at peaks of CNS pressure.

The observation that removing CSF from patients with symptomatic normal pressure hydrocephalus improves both arterial supply and venous drainage [19] supports the hypothesis proposed here. It is suggested that artificial increase in cranial pressure by balloon insufflation inside a lateral ventricle mimics the mechanism for progression of hydrocephalus. In this model, intracranial pressure is intermittently raised [20] in a manner that is comparable to that which will occur with movement in the presence of any cause of loss of compliance. Signs of a hyperemic response may have been observed during direct measurement of CNS pressure following pressure increase with movement in Chiari I [21]. Experimentally induced foramen magnum blockage has lead to subjective observations of venous insufficiency in the cord [22], and the effect of pressure on venous flow, causing localised oedema that resolves with improved perfusion of veins may be seen during surgery [23].

### Chiari malformation

Chiari I and II malformations are characterised by degrees of hindbrain herniation. Adults with Chiari I malformation have a reduced posterior fossa size, relative to head size [12,24-26], providing evidence for posterior fossa restriction as a cause of cerebellar herniation. Cerebellar herniation is a feature of posterior fossa restriction with known causes, for example craniosynostosis [27]. The familial tendency for Chiari I indicates that there are genetic determinants for posterior fossa growth [28,29]. Chiari II is characterised by a hypoplastic posterior fossa with compression of hindbrain structures [30]. Associated bone abnormalities in the face, provide additional evidence of a primary maldevelopment of bone as part of the malformation [30]. Facial and posterior fossa bones have common embryological origins and their responsiveness to fibroblast growth factor suggests that they have related growth mechanisms [31]. The greater incidence of Chiari I and II in females supports the view that posterior fossa size is genetically determined, with males having larger posterior fossa CSF spaces than females, so that restriction of posterior fossa size leads to hindbrain herniation more readily in females than males.

Spinal CSF and venous blood provide buoyancy to the central nervous system [9], maintaining the position of the hindbrain. The phenomenon of coning in response to lumbar puncture illustrates how hindbrain herniation can occur in response to loss of spinal fluid volume. The use of lumboperitoneal shunts is associated with cerebellar herniation [32], and is likely to be related to loss of CSF from the spinal compartment. Reduction of posterior fossa CSF space may also cause an abnormal cranio-spinal pressure gradient by retaining CSF in the head, which would otherwise be free to move through the foramen magnum. Direct measurement of pressure in the presence of Chiari I support the hypothesis that low CSF pressure may cause the cerebellar tonsils to be drawn into the spine [33]. It is suggested here that this may occur when venous blood leaves the spinal venous plexus so that excessively free drainage from the spinal plexus may also contribute to hindbrain herniation. In these instances, hindbrain symptoms may predominate over those caused by raised spinal pressure. In animal studies the hindbrain descends following creation of an artificial spina bifida lesion [34], and may elevate following intrauterine repair [34]. This allows for the common assertion that low spinal pressure contributes to the hindbrain herniation of Chiari II.

**Figure 3:**
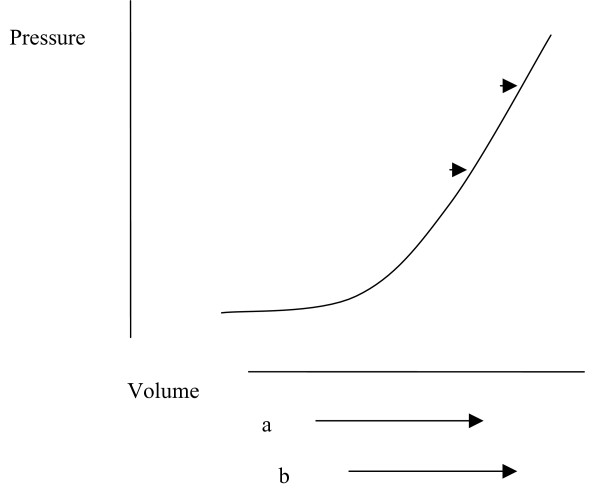
**Hypothetical graph showing the change in spinal volume and pressure with movement and a space-occupying lesion.** Lines a and b represent volume fluctuation with movement, with 'a' representing the normal range and 'b' the range with an uncompensated space occupying lesion. Points 'a' and 'b' on the curve indicate maximal CSF pressures that occur with maximal venous volume.

That the degree of hindbrain herniation does not correlate with spinal injury in syringomyelia [35] and spina bifida, and posterior fossa size does not correlate with the degree of cerebellar herniation in Chiari I [11,12,36], indicates that factors other than posterior fossa hypoplasia are influencing the degree of neural injury. Herniation will relate to the degree to which skeletal defects lead to pressure changes and the extent to which low spinal pressure causes hindbrain herniation in any individual case. Both herniation and neural injury will depend upon an interaction between the anatomy of the posterior fossa and physiological influences on CSF pressure and flow. Although low pressure may contribute to the cause of hindbrain herniation, once herniation becomes more established CNS pressure will tend to vary more widely as the head and spine become separate compartments. Pressure in the head may tend to rise [2] due to a larger volume of neural tissue in the head than the spine, with a greater arterial supply, as illustrated by the caudal flow of CSF across the foramen magnum in systole and the rostral flow in diastole [37]. The potential range in pressure is arguably greatest in the spine because of the capacity and compressibility of the intraspinal venous plexus vessels. This theory asserts that the highest spinal pressures lead to cord ischemia and the lowest pressures contribute to maintaining the hindbrain herniation. Loss of neural tissue by ischemic atrophy will tend to benefit remaining cord or brain, whereas growth of neural tissue may perpetuate reduced compliance. A syrinx cavity forms a space-occupying lesion and so reduces compliance. The effect of a space-occupying lesion on spinal pressure will be as in Fig. 3. Benign lesions may be accommodated without pressure increase if neural or CSF volumes fall. The combination of pressure effects illustrated in Figs. 2 and 3 will be a left shift in the pressure volume curve in hydrocephalus.

### Hydrocephalus

It is proposed that hydrocephalus is an oedema of the central nervous system. Rapidly developing venous insufficiency leads to increase in parenchyma water content and slower processes lead to increase in water that is distributed between the parenchyma and larger extracellular spaces. Symptoms, signs and morphology of hydrocephalus will depend upon the pathology that causes the pressure increase, the rate of fluid accumulation, and the developmental stage at which hydrocephalus develops. Compliance and therefore hydrocephalus, is influenced by:

• The internal dimensions of CNS bones

• Fontanelles and surgically created bone defects

• The presence of any space occupying lesion

• CSF volume

• Free flow of CSF around the CNS

• The integrity of CNS veins and central venous pressure

• Growth and atrophy of neural tissue

• Autonomic regulation of blood pressure and flow

Fluid flows down pressure gradients from areas of higher to lower pressure [1]. The passage of fluid into the ventricles and subarachnoid spaces represents flow to an area of lower mean pressure than the parenchyma. Maximal CSF pressures are achieved during physical movement and arterial pressures will tend to exceed this. Movement of tracers from the subarachnoid space to the parenchyma has been demonstrated to occur rapidly [38] and might appear to contradict this theory. If movement of fluid occurs along perivascular channels, such movement might depend upon the amplitude of the arterial pulse and the energy that it imparts [39]. According to the proposed hypothesis rapid accumulation of fluid would tend to remain in the parenchyma. Slow accumulation that occurs with intermittent periods of relaxation, when CSF pressure falls, would encourage fluid flow into CSF spaces.

Ventricle size is not a good indicator of intracranial pressure. Lateral ventricle size is determined by ventricle wall tension, brain turgor and the ability of CSF to flow through the aqueduct and fourth ventricle. Small ventricles may be found with raised, normal, or low intracranial pressure if CSF can exit the head [40]. Brain oedema may lead to small ventricles if CSF can pass into the spine. Fluid accumulation in the parenchyma will increase brain turgor and cause it to resist ventricle enlargement [41] whereas tension in the ventricle walls will exert a force on brain tissue, favouring enlargement of the ventricles. Wall tension is proportional to the internal radius of the cavity [10], as well as fluid pressure. Pressure waves generated by venous volume fluctuation in the spine may influence lateral ventricle wall tension without the requirement for flow [42], so physical movement may maintain or increase ventricle size when posterior fossa CSF pathways are patent. In chronic cases of raised intracranial pressure there will be atrophy related to ischemia, causing the brain to shrink. The posterior fossa CSF spaces and the aqueduct will remain open, giving the clinical presentation of normal pressure hydrocephalus. Rapid pressure increase will tend to cause obstructions to CSF flow. When the overall volume occupied by the brain increases with space occupying lesions, enlarged ventricles, or oedema, there may be obstruction of CSF flow at the aqueduct [43]. This is due to the narrowness of the aqueduct and its compressibility in comparison to the ventricles. As overall brain (including fluid) volume increases, CSF may be displaced from the subarachnoid spaces into the spine and the hindbrain may be displaced towards the foramen magnum. This may enhance the rate of intracerebral pressure increase by preventing the normal to and fro flow movement of CSF across the foramen magnum [44] that facilitates CNS venous drainage. These two major obstructive effects on CSF flow will contribute to the self-perpetuating nature of hydrocephalus, minor obstructions will also be detrimental.

A balance between brain turgor and ventricle pressure may occur, this is a feature of normal health and is also illustrated by cases where normal lateral ventricle size may occur with raised pressure and papilloedema. Alternatively, very rapid increase in ventricle pressure may cause symptoms of raised CNS pressure, with little time for the development of clinically detectable oedema of the optic nerve. A phenomenon of reactive enlargement of the ventricles following lumbar puncture has been observed in the presence of cerebral oedema [23]. In these cases a decrease in overall CNS pressure with lumbar puncture allows improved venous drainage, water passes out of the oedematous brain and CSF  may then move out of the spine into the cranial cavity as venous blood enters the spinal venous plexus. If the ventricles are small or obstructed and intracranial pressure is high the patient's condition will be critical. This will be indicated by obliteration of subarachnoid CSF spaces.

There are many disease processes that can lead to a state of CNS venous insufficiency by causing an increase in arterial supply, filtration of fluid into the parenchyma or venous pressure. An incomplete list includes CNS infection, hypoxia with altitude, carbon monoxide poisoning, water intoxication, renal impairment, obesity, and anaemia. Hydrocephalus may be caused by abnormalities of CSF production in the choroid plexus or absorption at the arachnoid granulations. That cardiac failure is an infrequent cause of hydrocephalus [45] may be an illustration of the efficiency of normal autoregulation of CNS extracellular fluid volume. Restriction of internal bone space through multiple suture craniosynostosis with a normally developing brain will tend to cause hydrocephalus [46] by compressing venous channels and CSF spaces. Single lambdoid suture fusion will be associated with raised pressure [46], because of the relative importance of posterior fossa size to ventricle emptying and foramen magnum flow. Direct and indirect measurements of venous pressure demonstrate variable correlation with hydrocephalus because pressure [5] and venous drainage [47] are dynamic processes. Current methods of quantifying these pressures are not representative of fluctuation with time.

Syringomyelia and spina bifida have been described as part of a disease continuum, with more severe manifestations in the fetus [48]. The mechanism for neural injury in the original theory had a requirement for hydrocephalus with CSF flow from the head into the cord parenchyma via the fourth ventricle. Hydrocephalus is not always present in the two conditions and fluid flow from the fourth ventricle into spinal cord cavities in syringomyelia is uncommon. It is proposed that the disease continuum relates to posterior fossa hypoplasia that causes reduced CNS compliance before or after birth.

### Anencephaly and Spina Bifida

Features of spina bifida that need to be addressed for a unifying hypothesis to be plausible include:

• The nature of progressive neural injury

• An association with hydrocephalus

• A higher incidence of Chiari malformation in females

• The relationship between the level and severity of the lesion

• Small head size in the fetus

• Intrauterine growth retardation

• Syringomyelia after birth

**Figure 4:**
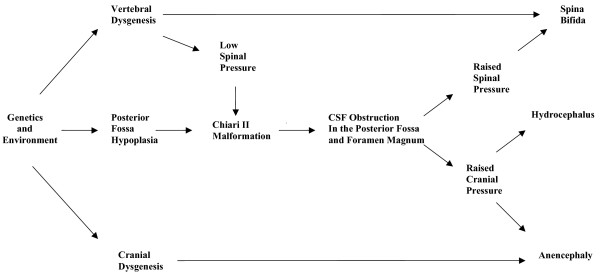
A simplified flow diagram illustrating the causes of spina bifida and anencephaly.

Animal models indicate that spina bifida may result from failure of neural tube closure [49]. Evidence suggests that mesodermal growth supports and shapes the neural tube to facilitate the closure process [49,50]. Vitamin A impairs growth of the mesoderm and may induce dysraphic disorders by this mechanism [51]. Bones of the basichondrium arise from mesoderm early in fetal development, prior to completion of neural tube closure [52]. Neural tube closure in humans proceeds in a rostral direction from the hindbrain region [49]. It is proposed that genetic and environmental influences have variable effects on mesodermal growth influencing posterior fossa size and the ability of the para-axial mesoderm, which forms vertebral bone, to facilitate neural tube closure. Growth reduction in the posterior fossa will tend to increase CNS pressure which opposes neural tube closure. A fine balance between growth and pressure may be required from the earliest stages to achieve normal development of central nervous system and surrounding bone. Where raised pressure interacts with mesodermal restriction a failure of neural tube closure will occur. A simplified representation of the mechanism for dysraphism is shown in Fig. 4.

The more severe the mesodermal impairment, the more hypoplastic the posterior fossa will tend to be, with earlier and greater potential for separation of the spinal and cerebral CSF spaces and subsequent neural injury. Anencephaly represents the highest and most severe lesion. A more normal posterior fossa size will lead to more normal cerebral development. If posterior fossa size is not significantly restricted and a small deficit in the vertebrae occurs, a meningocele will tend to result at any level. The effect of timed dosing of vitamin A in the creation of neural tube defects adds strength to the argument that abnormal mesodermal growth at different stages may result in different morphological features. This teratogen results in more frequent anencephaly when given early in gestation and more frequent spina bifida if dosing is delayed [53]. There will, according to this theory, be a tendency for higher lesions to be more severe, which accords with some observations on spina bifida [54,55].

In anencephaly and spina bifida, early neural development may be relatively normal [56], with progressive damage during gestation. Posterior fossa restriction occurs at an early stage with progressive hindbrain herniation [57,58]. It is suggested that any movement may cause mechanical force on neural tissue. Fetal cardiac movement is present from five post-menstrual weeks, trunk movements are detectable from seven weeks and the fetus is active by ten weeks [59]. It is proposed that disturbances in blood flow, accumulation of CSF and stretching forces act on tissue, which lacks mechanical support of the mesoderm. As fetal movement becomes stronger forces will be magnified around an open lesion, but the whole CNS may be affected. Widening of the whole vertebral column may be found in anencephaly, suggesting severe distending forces [48] and vertebral widening is found in the cervical spine with syringomyelia suggesting a mild distending force during growth [60].

**Figure 5:**
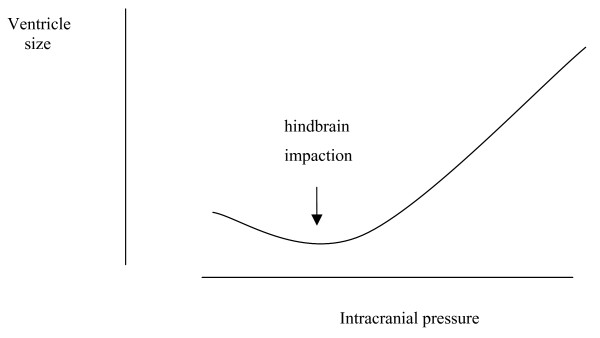
**Hypothetical graph depicting the relation between fetal lateral ventricle size and intracranial pressure and the effect of hindbrain impaction on ventricle size.** Hindbrain impaction is caused by a pressure gradient across the foramen magnum. A relatively small intracranial pressure gradient will cause the lateral ventricles to empty. Progressive increase in cerebral pressure will then cause the ventricles to enlarge.

Spina bifida is associated with reduced head size in the fetus [61]. This may be because as gestation progresses skull growth depends upon pulsatility of pressure. This view is supported by the rapidity of response to tensile forces in skull suture fibroblasts [62]. Pressure pulsations generated in the spinal venous plexus are normally transmitted to the head [13] but foramen magnum obstruction attenuates pressure transmission across the foramen magnum, between the two compartments [8,15]. Posterior fossa hypoplasia may, by compressing the hindbrain, block transmission of pressure pulsations and so reduce the normal stimulus for skull growth in the fetus. Pressure changes with movement will tend to be confined to the spine, dissipated by the vertebral defect and prevent the vertebral canal from closing. The lowest spinal pressures will occur during relaxation of the fetus and may be abnormally low or low for excessive periods, as a result of the vertebral defect. It is proposed that these mechanisms explains the majority of the progressive cord injury that is observed in spina bifida. Brain growth in the presence of skull restriction would tend to decrease ventricle size and worsen the hindbrain herniation until foramen magnum obstruction impairs fourth ventricle emptying sufficiently that the ventricles enlarge. The skeletal abnormalities and abnormal pressure gradient lead to a progressively worsening hindbrain herniation that tends to become moulded and impacted [63]. It is proposed that ventricle size in the affected fetus fluctuates with pressure according to Fig. 5. The phase of small ventricle size represents the early stage of relatively raised intracranial pressure. As hydrocephalus progresses the development of a severe foramen magnum obstruction is potentially lethal before or after birth.

Excessive pressure in the head or spine as a result of posterior fossa hypoplasia may be viewed as a progressive process occurring during or after gestation. In the fetus impairment to CSF flow may adversely influence neuronal migration [64]. Animal studies demonstrate that ischemia damages developing neural tissue and neurobehavioural abnormalities, such as memory deficits may result from such injury [65]. Abnormalities of migration have been described in human neurospheres transplanted into rat cerebral cortex following ischemic injury [66]. Such studies give clues as to the possible origin of complex cerebral abnormalities that may be found in anencephaly and spina bifida.

**Figure 6:**
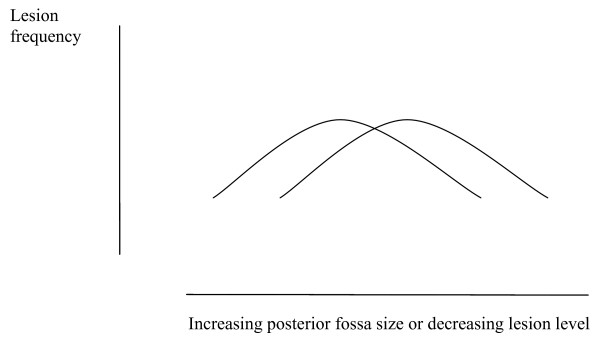
**Hypothetical graph showing the effect of posterior fossa size on the frequency of dysraphic lesions at different levels in males and females.** Higher, more severe, lesions tend to be more frequent in females who have a smaller posterior fossa as illustrated by the curve on the left. Lower, less severe, lesions are more frequent in males with a larger posterior fossa, as illustrated by the curve on the right.

The proposed hypothesis depends upon restriction of posterior fossa growth as a cause of neural injury with females having a smaller, genetically-determined, average posterior fossa size and cisterna magna CSF space than males. Posterior fossa size will be normally distributed. The genders represented separately will form overlapping curves with males to the right. If genetic or environmental factors that restrict growth of the posterior fossa interact with a normal variation in posterior fossa CSF space, and if the pathogenesis of spina bifida is related to hindbrain compression due to posterior fossa hypoplasia, there will tend to be a difference in severity of lesions between the sexes. Anencephaly will predominate in females, with lower spinal lesions being more common in males. This concept is represented in Fig. 6, and accords with observations on differences between lesion prevalence between the genders [49,67]. This hypothesis allows speculation that smaller CSF spaces in the female are part of a regulatory mechanism leading to smaller head size. With similar fluctuations in spinal venous volume a more spacious posterior fossa in the male may facilitate greater pressure wave transmission into the head resulting in larger fetal head size [68].

Acute worsening of obstructive hydrocephalus after birth has been observed with spina bifida [69]. Repair of the spinal lesion may increase CNS pressure, whereas loss of CSF from a lesion may be beneficial for the fetus as gestation progresses in circumstances where CSF tends to accumulate. Breathing air and expansion of the thorax will increase capacitance in pulmonary vessels and reduce thoracic pressure [70]. This will tend to improve CNS venous drainage and lower CNS pressure. Improved venous drainage may contribute to the low CSF pressure found in normal neonates [71]. It may also encourage herniation of the hindbrain due to lowering of pressure in the spinal venous plexus. Loss of brain turgor associated with weight loss that tends to occur in the neonatal period [71] may also contribute to ventricle enlargement. These factors in combination will tend to favour foramen magnum obstruction, whereas taping of CSF in the neonatal period may avert an obstruction to CSF flow.

Abdominal growth tends to be reduced in fetuses with open spina bifida as gestation progresses [61]. If degrees of CNS hypoxia are a feature of spina bifida that also progresses during gestation then it is possible that this pattern of abnormal abdominal growth would be expected. One mechanism for accommodating a chronic increase in central nervous system pressure may be decreased cardiac output [72] suggesting a mechanism for growth retardation. Areas of brain that are most damaged in anencephaly are also the most susceptible to ischemia in chronic hydrocephalus [72,73]. The relative preservation of basal brain structures in anencephaly will relate to their blood supply. Cervical meningoceles are sometimes found, but are not associated with significant CSF obstruction at the foramen magnum and neural development appears to be normal [74]. Support for a theory of ischemic damage in spina bifida has been obtained from histological observations on the spine, movement analysis of neonates with spina bifida, [75,76] and the presence of metabolites associated with ischemia in the CSF of affected neonates [77].

### Syringomyelia

**Figure 7:**
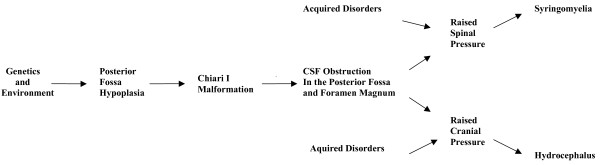
A simplified flow diagram representing the causes of hydrocephalus and syringomyelia.

Surgery that improves CSF pathways at the foramen magnum may cause collapse of the syrinx cavity, improve cord blood flow and the clinical features of syringomyelia. This indicates that neural injury results from impaired flow of CSF at the foramen magnum [1,2]. The central canal of the cord forms a potential space, into which fluid from the parenchyma may pass, allowing the formation of separate cavities that characterise syringomyelia [78]. During physical exertion, movement of fluid within the syrinx cavity may weaken its walls causing it to have a greater capacity to enlarge [79,80] and during relaxation, the cavity will tend to fill. This may be facilitated by a hyperaemic response. Syringomyelia occurs in association with posterior fossa restriction of Chiari I and other causes of reduced CNS compliance, particularly if they directly affect the spine. Tumours, vertebral deformity and arachnoiditis may reduce space and CSF flow and may lead to the onset of cavity formation. Vertebral injury causing abnormal function of the spinal venous plexus may impair spinal venous drainage as a result of autonomic dysfunction [81]. Decompressing the cord ameliorates syringomyelia, and reducing the volume of fluid inside a syrinx cavity at operation may enhance the rate of any post-operative improvement [82]. Normal pressure hydrocephalus is an analogous disease process to syringomyelia, as has been argued elsewhere, [83] with differing distribution of excess extracellular fluid. Spinal cord oedema would, according to this theory, represent a pre-syrinx state [84]. Syringobulbia is the manifestation of raised spinal pressure with the unusual situation of fluid being able to track into the brain stem. Communicating syringomyelia would occur with pressure gradients and anatomy allowing flow from the fourth ventricle into the spine. A simplified representation of the mechanism for hydrocephalus and syringomyelia is shown in Fig. 7. Posterior fossa hypoplasia makes hydrocephalus and syringomyelia features of spina bifida after birth.

## Conclusion

A hypothesis to unify all causes of hydrocephalus is possible by considering the effects of physical movement on CNS pressure when compliance is reduced. The hypothesis argues that hydrocephalus is a self-perpetuating problem caused by loss of compliance and also causing loss of compliance by the accumulation of excessive extracellular fluid which may obstruct CSF flow. The hypothesis allows for the suggestion that a combination of cranial expansion and relief of obstruction to CSF flow, including plastic surgery to the posterior fossa, may reduce the necessity for some shunt procedures. Continuous pressure monitoring during activity causing reversible ischemia would provide direct evidence for the proposed mechanism of hydrocephalus and if this predicted effect could be demonstrated, tests for reversible ischemia may be of diagnostic use. An absence of reversible ischemia would suggest a compensated phase of the disease lacking the potential for a favourable surgical outcome.

Chiari malformations are primarily caused by congenital posterior fossa hypoplasia sufficient to cause obstruction to CSF flow, which damages the CNS. Abnormal mesodermal growth leads to abnormalities of central nervous system pressure. The manifestations of Chiari related syringomyelia, spina bifida and anencephaly form a spectrum of disease. If fetal posterior fossa CSF flow can be improved there is the potential for reducing the impact of Chiari malformation before birth.

## Competing interests

The author(s) declare that they have no competing interests.

## Authors' contributions

This is the work of one author. The author has read and approved the final version of the manuscript.

## Supplementary Material

Additional file 1 Permission to redraw Fig. 3 from [5]. The CNS pressure volume curve. The curve has three zones, a flat zone expressing good compensatory reserve, an exponential zone, depicting poor compensatory reserve and a final zone seen at very high ICP depicting derangement of normal cerebrovascular responses. In [5] it is shown that pulse amplitude increases linearly with mean intracranial pressure in the zone of poor compensatory reserve. The graph is redrawn to suggest that this phase corresponds to a phase of reduced compliance when pulsation caused by blood flow under the influence of autoregulation is detectable. At low pressure pulsations are less evident because of compliance and at the highest pressure pulsatility decreases as arterial and venous flow is compromised. Click here for file
