# Supplementary figures and images for: A unifying hypothesis for hydrocephalus, Chiari malformation, syringomyelia, anencephaly and spina bifida
Source: Cerebrospinal Fluid Res. 2008 Apr 11;5:7. doi: 10.1186/1743-8454-5-7 (PMC2365936; doi:10.1186/1743-8454-5-7)

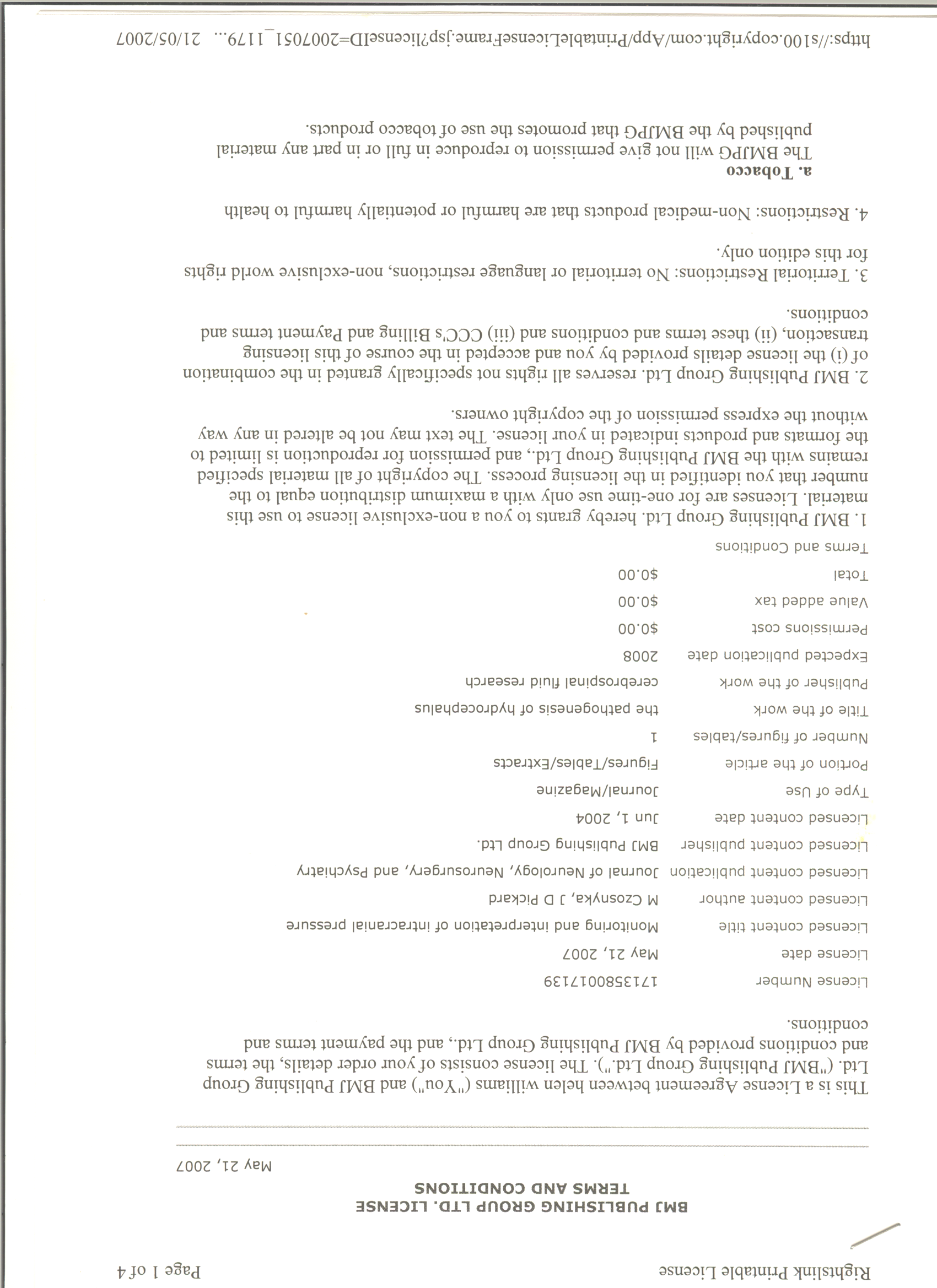

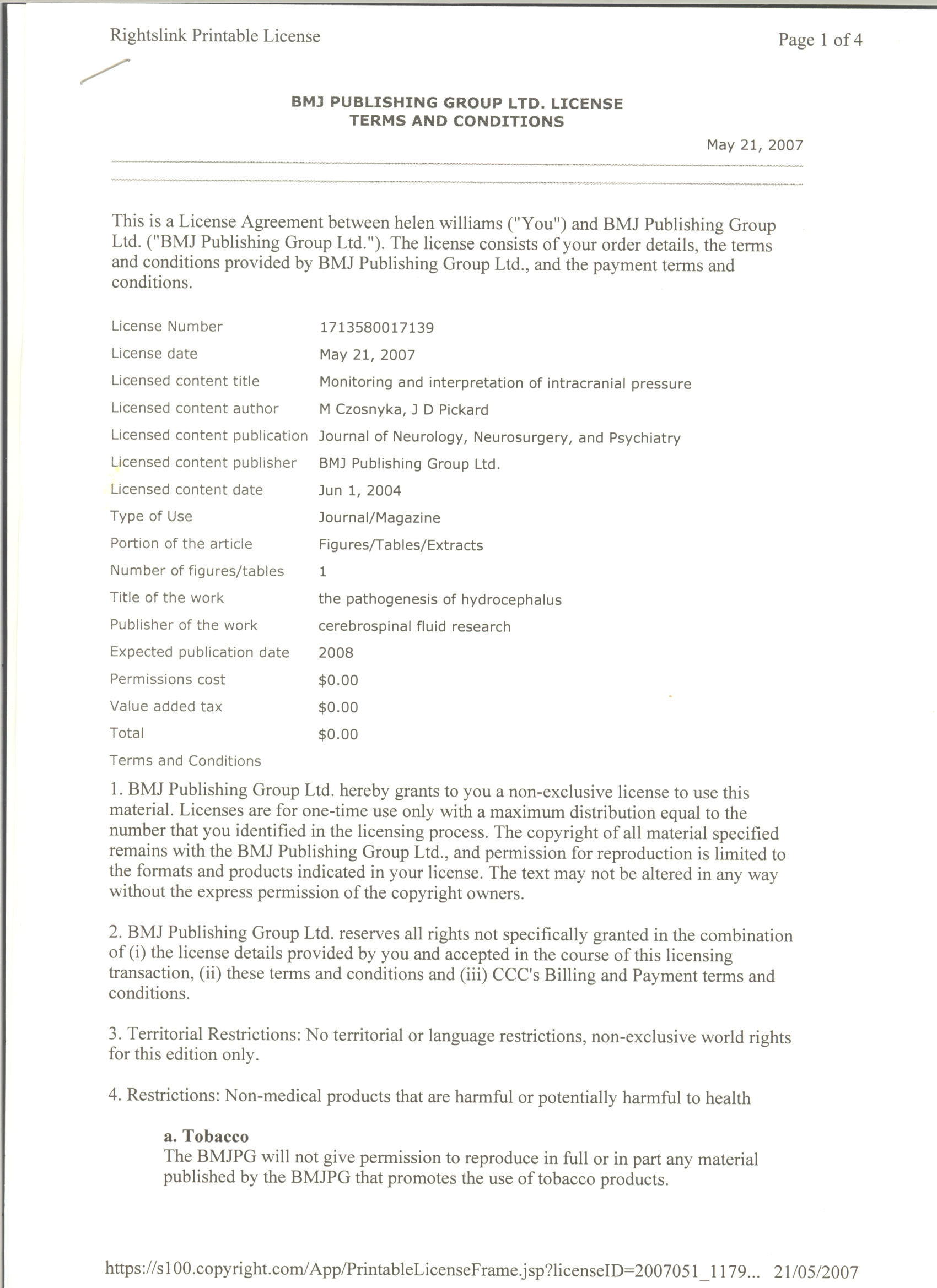

Supplement: Additional file 1 — Permission to redraw Fig. 3 from [5]. The CNS pressure volume curve. The curve has three zones, a flat zone expressing good compensatory reserve, an exponential zone, depicting poor compensatory reserve and a final zone seen at very high ICP depicting derangement of normal cerebrovascular responses. In [5] it is shown that pulse amplitude increases linearly with mean intracranial pressure in the zone of poor compensatory reserve. The graph is redrawn to suggest that this phase corresponds to a phase of reduced compliance when pulsation caused by blood flow under the influence of autoregulation is detectable. At low pressure pulsations are less evident because of compliance and at the highest pressure pulsatility decreases as arterial and venous flow is compromised. [file 1743-8454-5-7-S1.doc]
